# Social, Environmental and Behavioral Determinants of Asthma Symptoms in Brazilian Middle School Students—A National School Health Survey (Pense 2012)

**DOI:** 10.3390/ijerph15122904

**Published:** 2018-12-19

**Authors:** Rita C. Ribeiro-Silva, Deborah C. Malta, Laura C. Rodrigues, Dandara O. Ramos, Rosemeire L. Fiaccone, Daiane B. Machado, Maurício L. Barreto

**Affiliations:** 1 School of Nutrition, Federal University of Bahia, Salvador 40.110-150, Brazil; 2 School of Nursery, Federal University of Minas Gerais, Belo Horizonte 30.130-100, Brazil; dcmalta@uol.com.br; 3 Department of Infectious Disease Epidemiology, London School of Hygiene and Tropical Medicine, London WC1E 7HT, UK; laurarodriguesinlondon@gmail.com; 4 Center for Data and Knowledge Integration for Health (CIDACS), Institute Gonçalo Moniz (IGM), Oswaldo Cruz Foundation (FIOCRUZ), Salvador 41.745-715, Brazil; dandararamos2@gmail.com (D.O.R.); daianedbm@hotmail.com (D.B.M.); mauricio@ufba.br (M.L.B.); 5 Institute of Mathematics and Statistics, Federal University of Bahia, Salvador 40.170-110, Brazil; rose.fiaccone@gmail.com; 6 Institute of Collective Health (ISC), Federal University of Bahia, Salvador 40110-040, Brazil

**Keywords:** wheezing, schoolchildren, psychosocial indicators, violence, lifestyle

## Abstract

Biological and psychosocial factors are recognized contributors to the worldwide burden of asthma. However, the relationship between psychosocial factors and asthma symptoms among students in low- and middle-income countries remains underexplored. We aimed to identify socioeconomic, environmental, psychosocial, family-related and lifestyle factors associated with the self-reporting of asthma symptoms in Brazilian adolescents. This is a cross-sectional study using data from the 2012 PeNSE survey (*n* = 109,104). We analyzed the following variables: socioeconomic conditions, demographic characteristics, lifestyle, family context and dynamics, psychosocial indicators, smoking, and exposure to violence. Our outcome variable was the self-report of asthma symptoms in the past 12 months. The prevalence of wheezing was 22.7% (21.5–23.9). After adjusting for sex, age and the variables from higher hierarchical levels, exposure to violence (feeling unsafe at school, being frequently bullied, being exposed to fights with firearms) and physical aggression by an adult in the family were the environmental factors that showed the strongest associations with self-reporting of asthma symptoms. For psychosocial indicators of mental health and social integration, feelings of loneliness and sleeping problems were the strongest factors, and among individual behavioral factors, the largest associations were found for tobacco consumption. Our findings were consistent with previous studies, showing an association between self-reported asthma symptoms and socio-economic status, family context and dynamics, psychosocial indicators of mental health, exposure to violence and social integration, as well as a sedentary lifestyle and tobacco use.

## 1. Introduction

Asthma is a common long-term inflammatory disease of the airways of the lungs. It is characterized by chronic inflammation associated with the hyper-responsivity of the airways, leading to recurrent episodes of wheezing, coughing, chest tightness, and shortness of breath, particularly in the early morning or at night [1].

Asthma is the 14th most important disorder in the world according to years lived with a disability. It is estimated that approximately 334 million people around the world have asthma [2]. The burden of asthma, measured by disability and premature death, is greatest among children (10–14 years old) and the elderly (75–79 years old) [2]. Current studies have estimated that 14% of children throughout the world experience asthma symptoms [2]. Epidemiologic studies, such as the International Study of Asthma and Allergies in Childhood (ISAAC), registered an average world prevalence of 14.1% for asthma symptoms in adolescents between the ages of 13 and 14, including 5.1% in Northern and Eastern Europe, 22% in Oceania, and 15.9% in Latin America [3]. Most individuals affected by asthma live in low- and middle-income countries, and its prevalence is increasing at the fastest rate in these countries [2]. In Brazil, the asthma prevalence is one of the highest in Latin America, estimated at 12% for the adult population [4], and between 13% [5] and 23% among children and adolescents [6]. Official data, from government sources, indicates that asthma is still one of the twenty most common reasons for primary care visits in Brazil, representing the third leading cause of hospitalization within the Brazilian public health system (SUS—Sistema Único de Saúde) [7].

Some important risk factors for asthma in children and adolescents have been identified; most of these risk factors are related to a family history of asthma and asthma symptoms, rhinitis or eczema [8], excess humidity at home and infectious respiratory diseases during childhood [9], indicators of poverty or a low socioeconomic status (SES), such as low maternal education and lack of cleanliness at home [10], nutritional status [11,12], psychosocial context [13], sedentary lifestyle [14], factors related to pregnancy and perinatal conditions [15] and genetic factors [16,17]. While a large number of common risk factors have been assessed, these factors are insufficient to explain the development of a multifactorial disease such as asthma. Efforts to define the etiological risk factors for the development and expression of asthma have intensified due to observed increases in the prevalence and severity of asthma throughout the world.

Recent studies have highlighted a complex network of interactions among economic, psychosocial and environmental factors [18]; the inclusion of environmental factors provides us with fresh insight into how various stressors may influence the development of diseases. Epidemiological studies have demonstrated associations [19,20] among stress, social status, quality of social relationships—social networks and an individual’s ties to friends, family, work, and community through social and religious groups—and health. These findings led to a paradigm shift that reconsidered the overlap between biological determinants and psychosocial factors in the rising asthma burden. Other studies [21,22] have pointed out associations between stress and psychological factors with asthma symptomatology and bronchoconstriction in the reduced pulmonary flow rates in asthmatic children [23]. These data support an association with stress, although the causal mechanisms linking stress and asthma in children are still unclear.

Biological and psychosocial factors are increasingly recognized contributors to the worldwide burden of asthma. However, the relationship between psychosocial factors and asthma symptoms among students in low- and middle-income countries remains unexamined. We evaluated socioeconomic, environmental, psychosocial, family-related and lifestyle factors associated with the self-report of asthma symptoms in Brazilian adolescents, drawing on the PeNSE survey (Pesquisa Nacional de Saúde do Escolar/National Adolescent School-Based Health Survey), which was conducted in 2012 by the Ministry of Health, Brazil, in collaboration with the Brazilian Institute of Geography and Statistics (IBGE).

## 2. Materials and Methods 

### 2.1. Study Design, Population and Data Collection

This is a cross-sectional study using data from the 2012 edition of the PeNSE survey. The PeNSE sample was chosen to represent the population of 9th grade students in Brazil, including its five regions, 26 state capitals and the Federal District (*n* = 109,104). For the sampling plan, the national territory was stratified as follows: each of the 26 state capitals and the Federal District were defined as a geographic stratum, and the other cities were grouped in each of the five major geographic regions, thus forming another five geographic strata. The sample from each stratum was proportional to the number of schools, according to the administrative dependency of the schools (private and public). For each stratum, a conglomerate sample was selected in two stages, as follows: in the first stage, schools were selected; in the second stage, eligible classrooms were chosen from the selected schools (9th grade, elementary school).

In the strata formed by cities that were not capitals, conglomerates were grouped according to homogeneity and neighboring criteria, thus forming groups of approximately 300 to 600 classrooms. One sample of these groups was selected from each region, and afterwards, schools were selected. Thus, the primary sampling units were the groups of cities and the secondary units were schools; classrooms from these schools were the tertiary sampling units. In both cases, all students in the selected classrooms who were present on the day of data collection formed the sample of students who were invited to participate in the study. For further details on the sampling strategy see PeNSE, 2012 [24]. 

The 9th grade was chosen because these students, mostly between 13 and 15 years old, have acquired the necessary skills to answer the self-applied structured questionnaire and are likely to be exposed to several risk factors; also, the data from this population enable a comparison with other countries [25]. The questionnaire, comprising approximately 120 questions, was installed on a smartphone. 

Two questions about asthma were included [24]: “In the past 12 months, did you have wheezing (or chirping) in your chest?” (yes/no) and “Have you ever had asthma?” (yes/no).

The PeNSE survey was approved by the Ethics Research Committee of the Brazilian Ministry of Health, report NO. 192/2012, concerning registration NO. 16805, of CONEP/MS, on 27/03/2012.

### 2.2. Measures

Socioeconomic conditions: skin color (white (reference), black, brown, other), mother’s educational level (no education or unfinished primary education (reference), primary education or unfinished intermediate education, intermediate education or unfinished higher education, higher education), father’s educational level (no education or unfinished primary education (reference), primary education or unfinished intermediate education, intermediate education or unfinished higher education, higher education), school’s administrative status (private (reference), public), and school’s location (capital (reference), outside the capital). A categorized indicator of the person’s socioeconomic status (SES) was created according to educational level of the head of the household, possession of various types of household goods (e.g., television sets, desktop or laptop computer), vehicles and number of housekeepers.

Demographic characteristics: age ((<14 years (reference), 14 years, 15 years, >15 years)), (Gender (boys, reference), girls)).

Lifestyle: daily time of sedentary leisure (≤4 h sitting watching TV, etc./day (reference), >4 h sitting watching TV etc./day), and daily time spent watching TV (≤2 h/day(reference), >2 h/day).

Family context and dynamics: presence of parents/guardians at home (presence of mother and father (reference), only mother present, only father present, absence of mother and father), parents/guardians acquainted with the leisure activities of their child ((rarely/never, frequently (reference)), parents/guardians attending meals at home (rarely/never, frequently (reference)), parents/guardians checking their child’s homework ((rarely/never, frequently (reference)), and understanding of the child’s problems by the parents/guardians (rare/never, frequent (reference)), parents/guardians rummaging without permission in their child’s personal belongings (rarely/never (reference), frequently).

Psychosocial indicators: feeling of loneliness (rare/never (reference), frequent), loss of sleep due to concerns (rare/never (reference), frequent), existence of close friends (less than 3 friends, 3 or more friends (reference)), and child’s truancies (rare/never, frequent (reference)).

Smoking: personal tobacco use (yes, no (reference)) and tobacco use in the family (none of the parents/guardians (reference), at least one).

Exposure to violence: being the victim of bullying (rare/never (reference), frequent), feeling of security on the way from home to school (yes (reference), no), feeling of security at school (yes (reference), no), physical aggression committed by an adult family member (yes, no (reference)), and exposure to fights with firearms (yes, no (reference)).

Our outcome variable was the self-report of asthma symptoms in the past 12 months (“In the past 12 months, did you have wheezing or chirping in your chest?” Yes/No).

### 2.3. Statistical Analysis

**Figure 1:**
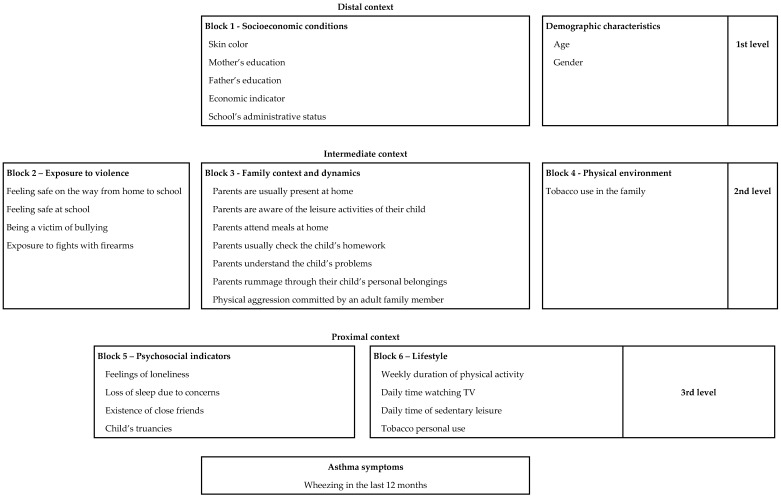
Hierarchical Conceptual Model of Risks Associated with Asthma Symptoms in Brazilian Middle School Students (PeNSE Survey, 2012).

Analysis was conducted following a predefined conceptual model similar to the framework suggested by Victora et al. [26]. This framework maps the proposed relationships between asthma symptoms and socioeconomic, family-related, individual behavior-related and psychological factors, which are organized in blocks and distributed among three levels (Figure 1). In this way, we implemented a hierarchical effect decomposition (HED) strategy to quantify the association of the factors on the different levels and to disentangle the direct and indirect associations of these factors. According to (HED), the poisson regression (PR) refers to the ‘overall effect’, i.e., the association adjusted for all potential confounding variables from the same or upper levels of the conceptual framework. For instance, the overall association of block 2 was adjusted for age, gender and SES (located one association level above) and block 3 (located on the same level).

First, tri-variate Poisson regression models were carried out to identify potential asthma determinants by calculating the sex- and age-adjusted prevalence ratios of asthma symptoms in exposed vs non-exposed children. The explanatory variables that, in these initial models, produced a *p*-value ≤ 0.10 were admitted to the next step, which was an intra-block backward selection, and only covariates with *p*-values ≤ 0.05 at this second step were included in the multivariate analysis of hierarchical modeling.

Imputation was used for a small subset of the data, regarding the variables composing our SES indicator. The variable informing socioeconomic status (SES) was created according to educational level of the head of the household, possession of various types of household goods (e.g., desktop or laptop computer), vehicles and number of housekeepers. As there was a considerable amount of absence of information regarding maternal educational level (17%) and also educational level of the father (23%), we imputed the mode for these categorical variables, stratifying according to the school’s location and administrative status, sex and age of the participant. Sensitivity analysis was performed by comparing the results of tri-variate Poisson regression models without and with imputation of the explanatory variables.

All statistical analyses were carried out using the statistical software package STATA (version 9.2, STATA Corporation, College Station, TX, USA), and weighted data analyses were conducted using the ‘svy’ family of commands to adjust the prevalence and variance estimates to account for the sample design and clustering.

## 3. Results

The prevalence of wheezing in the sample was 22.7% (21.5–23.9). Table 1 shows the descriptive of the sample and also bivariate associations with self-reports of asthma symptoms. Estimates of PR (prevalence ratio) and the 95% CI from Poisson multivariate model fitted are shown at Table 2.

In Table 1, the results show that after adjusting for sex and age among the block 1 variables (socioeconomic conditions), only black participants’ skin color showed no significant association with a self-report of asthma symptoms. All variables from blocks two, three, four, five and six were associated with a self-report of asthma symptoms in this first step of the analysis sequence.

Table 2 shows the additional two steps of the analysis. In step 2, the intra-block regressions of the asthma symptoms for the variables that had *p*-values ≤ 0.10 in the tri-variate analysis (step 1) were adjusted by sex, age and all of the variables in the block. In step 3, the regression analysis of the asthma symptoms was adjusted by age, sex and the variables that had *p*-values ≤ 0.05 in step 2, with further exclusion of the variables that had *p*-values > 0.05 according to the intra-level analysis.

For block one, the parents’ educational level was omitted from the intra-block analysis as being part of the SES indicator. The results from this block alone showed an increased prevalence of asthma symptoms among high SES participants and no significant association with the school administrative status. For blocks two, three, four, five and six, intra-block analysis showed an increased prevalence of reports of asthma symptoms among participants who also reported feeling unsafe at school (PR = 1.37; CI = 1.30–1.45; *p* < 0.001) or on the way to/from school (PR = 1.19; CI = 1.14–1.25; *p* < 0.001), being frequently bullied (PR = 1.36; CI = 1.29–1.44; *p* < 0.001), being exposed to fights with firearms (PR = 1.63; CI = 1.55–1.70; *p* < 0.001), lacking parental participation in everyday activities (meals (PR = 1.11; CI = 1.06–1.51; *p* < 0.001), rarely or never having their homework checked (PR = 1.13; CI = 1.08–1.19; *p* < 0.001), feeling understood (PR = 1.19; CI = 1.13–1.25; *p* < 0.001), experiencing parental intrusiveness (rummaging through their personal belongings without permission) (PR = 1.18; CI = 1.13–1.24; *p* < 0.001), being a victim of physical aggression by an adult family member (PR = 1.61; CI = 1.56–1.67; *p* < 0.001), tobacco use in the family (PR = 1.12; CI = 1.07–1.18; *p* < 0.001), feelings of loneliness (PR = 1.38; CI = 1.33–1.43; *p* < 0.001), sleep difficulties (PR = 1.37; CI = 1.33–1.41; *p* < 0.001), having less than three friends (PR = 1.15; CI = 1.10–1.19; *p* < 0.001), truancies (PR = 1.45; CI = 1.34–1.57; *p* < 0.001), having more than 4 h of sedentary leisure (PR = 1.17; CI = 1.13–1.21; *p* < 0.001) and being a tobacco user (PR = 1.53; CI = 1.45–1.61; *p* < 0.001).

In the final model, after adjusting for sex, age and the variables from the higher hierarchical levels, exposure to violence (feeling unsafe at school, being frequently bullied, being exposed to fights with firearms) and physical aggression by an adult in the family were the environmental factors that showed the strongest associations with a self-report of asthma symptoms; as for psychosocial indicators of mental health and social integration, feelings of loneliness and sleeping problems were the strongest factors, and among individual behavior factors, the largest associations were found for tobacco consumption.

## 4. Discussion

To our knowledge, our study is the first to examine the determinants of asthma symptoms using a large, nationally representative sample of adolescents in Brazil. We quantified the magnitude of the relative mediated risk of these factors using a HED strategy based on a predefined conceptual framework. The results from PeNSE indicate the high prevalence of asthma symptoms (23.2%) among 9th grade (elementary school) students, who are usually aged between 13 and 15 years old. This high prevalence was also observed in other regions of the world, such as North America (21.5%), Latin America (18.8%) and Oceania (26.7%) [27]. The high prevalence of asthma symptoms in the last 12 months among school children highlights asthma as an important health problem among adolescents and demonstrates that Brazil is among the countries with the highest prevalence of asthma in the world. Our findings were consistent with previous studies that showed an association of wheezing with socio-economic status [10,28], family context and dynamics [19,20], psychosocial indicators of mental health and social integration [13,21,22], sedentarism [14], and tobacco consumption [29]. Our study adds information about the association of asthma with violence among children in Brazil.

Among the variables explored in our model, those related to violence and physical aggression showed the strongest associations with asthma symptoms, consistent with previous studies [30,31,32] and suggesting that violent life events in childhood and adolescence are linked to a greater prevalence of self-reported asthma symptoms. The literature suggests that stress mediates the link between violence and asthma symptoms [32,33,34,35]. The oxidative stress hypothesis could explain the relationship among stress, hypothalamic–pituitary–adrenal (HPA) axis alterations, and the immune system [34]. Evidence also suggest that families living in more violent communities keep their children at home for longer periods of time, thus favoring contact with indoor allergens and triggering asthma attacks [36]. In addition, communities with heavier exposure to violence have limited access to health services, thus contributing to the severity of the asthma symptoms [37]. Because of the cross-sectional nature of the data, a plausible alternative explanation for our findings is that children and adolescents with asthma are more likely to be victims of bullying and violence than those who are healthy. However, regardless of the causes of the determined association, our findings highlight the importance of both screening for diseases such as asthma in children and adolescents who have been abused and of being aware of the possibility of abuse in children with asthma.

The association between psychosocial problems and the self-report of asthma symptoms found in our study demonstrated that the prevalence was greater among children who also reported feelings of loneliness and sleep difficulties and had fewer than three friends as well as children with truancies, with the strongest associations for self-reported feelings of loneliness and sleeping problems. These results are in line with the literature on the relationship between child mental health and asthma [13,35,38]. Studies have examined the relationship between child mental health problems and chronic diseases; however, the results have been controversial [39]. Although studies have shown heterogeneous effects between behavioral/psychosocial factors and asthma in the literature, a meta-analysis suggested that children with asthma have more behavioral difficulties than healthy children, with the effect of internalizing behaviors being greater than that of externalizing behaviors [40]. The co-occurrence of behavioral problems and asthma has been associated with lower adherence to treatment and an increased frequency and length of hospitalization episodes [41,42].

We also found that tobacco was associated with asthma symptoms. The body of evidence confirming the association between tobacco consumption and respiratory disease is substantial and unequivocal [43]. Previous laboratory studies in humans and animals suggest that nicotine is the component that has the most significant detrimental effects on lung growth and collagen deposition. Nicotine affects lung branching through stimulation of alpha-7 nicotinic acetylcholine receptors during the pseudo-glandular phase, resulting in disynaptic lung growth. Changes in the conducting airway structure can decrease airflow and increase resistance, decreasing pulmonary function [43,44]. Smoking status is also an important factor because smoking is associated with poor asthma control and asthma exacerbation; additionally, smokers are more likely to visit the emergency room for asthma and have trouble sleeping due to asthma symptoms [45].

### Limitations

The limitations of this study were primarily due to study design. Indeed, we emphasize that this cross-sectional study cannot establish a causal relationship due to the temporal sequence between exposure and effect. Also, precision of confidence intervals for SES indicator can be affected due to our imputation approach once parameter estimates may be biased. Triggering of asthma following stressful events has been documented [27,46], however, since the data collection was self-reported, the answers for psychosocial events might have been influenced by the students’ understanding of the topic. Also, adolescents’ reports of asthma symptoms may be imprecise, given their erroneous interpretations of the perceptions of these symptoms, as well as problems understanding the questions. Difficulties were reported by adolescents in understanding the term “wheezing”, as used in the ISAAC questionnaire. However, in Brazil, a validation study showed that wheezing in the past 12 months had high sensitivity, specificity and positive and adverse predictive value, reinforcing the concept that this is the key question for the diagnosis of asthma [47].

## 5. Conclusions

Our findings were consistent with previous studies, which have shown an association between the self-report of asthma symptoms and socio-economic status, family context and dynamics, psychosocial indicators of mental health, exposure to violence and social integration, as well as sedentary lifestyle and tobacco use. The results of this study can help managers to develop useful public health interventions in developing countries where there is no public strategy to prevent asthma. An understanding of the multiple factors associated with asthma morbidity, including the increased risk resulting from exposure to violence, will thus lead to the inclusion of psychological measures in the planning of health and childcare services for asthmatic children.

## Figures and Tables

**Table 1:** Pense 2012—Asthma Symptoms in Brazilian Middle School students: Characteristics Studied and Asthma Symptoms.

Variables	*n*	%	Asthma Symptoms					
			*n*	% ^1^	95% CI	PR ^2^	95% CI	*p*-Value
	109,104		25,038	22.7	21.5–23.9			
**DEMOGRAPHIC CHARACTERISTICS**								
**Gender**								
Boys	52,015	47.7	11,062	20.7	18.9–22.7			
Girls	57,089	52.3	13976	24.4	23.3–25.4	1.17	1.08–1.27	<0.001
**Age**								
<14 years old	22,443	20.6	5174	23.3	21.9–24.9			
14 years	50,900	46.7	11,834	23.1	21.9–24.4	1.00	0.96–1.04	0.10
15 years	21,105	19.3	4769	22.2	20.9–23.6	0.97	0.93–1.01	
16 years or older	14,656	13.4	3261	21.0	20.0–22.0	0.92	0.85–1.00	
**SOCIOECONOMIC CONDITIONS**								
**Self-referred skin color**								
White	37,674	34.6	8699	23.0	21.4–24.6			
Black	14,513	13.3	3259	21.8	19.4–24.4	0.97	0.89–1.05	0.61
Brown	48,237	44.2	10,976	22.6	21.9–23.2	0.98	0.93–1.03	
Others	8611	7.9	2104	23.4	20.8–26.1	1.02	0.95–1.08	
**Mother’s education**								
No education or primary education unfinished	33,322	36.8	7522	22.4	21.7–23.2			
Primary education, or intermediate education unfinished	15,975	17.6	3682	23.0	21.2–24.9	1.02	0.98–1.07	<0.001
Intermediate education, or higher education unfinished	28,244	31.2	6769	23.7	22.3–25.2	1.09	1.05–1.13	
Higher education	13,036	14.4	3255	25.5	24.2–26.9	1.17	1.10 –1.25	
**Father’s education**								
No education or primary education unfinished	35,174	41.9	7982	22.4	21.1–23.8			
Primary education, or intermediate education unfinished	14,114	16.8	3300	23.4	21.7–25.2	1.01	0.94–1.08	<0.001
Intermediate education, or higher education unfinished	22,617	26.9	5366	23.4	21.3–25.7	1.06	0.96–1.16	
Higher education	12,119	14.4	3049	25.9	24.7–27.1	1.17	1.12–1.23	
**SES indicator**								
1st tertile + 2nd tertile (D + C class)	50,430	64.4	5867	24.0	23.1–24.9			
3rd tertile (B class)	27,868	35.6	7048	25.1	23.2–27.1	1.16	1.10–1.23	<0.001
**School’s administrative status**								
Public	86,599	79.4	19,495	22.3	21.1–23.5			
Private	22,504	20.6	5543	25.0	24.1–26.0	1.12	1.05–1.19	<0.001
**EXPOSURE TO VIOLENCE**								
**Feeling safe on the way from home to school**								
Yes	99,497	91.6	21,899	21.7	20.4–23.0			
No	9150	8.4	3113	33.1	31.0–35.4	1.55	1.49–1.62	<0.001
**Feeling safe at school**								
Yes	100,126	92.4	22,004	21.5	20.4–22.7			
No	8187	7.6	2924	35.6	32.7–38.7	1.69	1.60–1.78	<0.001
**Being victim of bullying**								
Rarely/never	101,310	93.4	22,612	21.9	20.7–23.2			
Frequently	7193	6.6	2350	32.8	31.3–34.3	1.51	1.42–1.61	<0.001
**Exposure to fights with firearms**								
No	96,771	89.3	20,912	21.2	20.0–22.5			
Yes	11,648	10.7	4053	35.6	34.3–36.9	1.76	1.69–1.85	<0.001
**FAMILY CONTEXT AND DYNAMICS**								
**Parents are frequently present at home**								
Presence of mother and/or father	101,845	93.5	23,221	22.5	21.2–23.8			
Absence of mother and father	7065	6.5	1775	25.4	24.3–26.5	1.13	1.04–1.23	0.004
**Parents are aware of the leisure activities of their child**								
Frequently	63,916	58.9	13,815	21.3	20.3–22.4			
Rarely/never	44,656	41.1	11,168	24.5	23.0–26.1	1.17	1.14–1.20	<0.001
**Parents are present during meals**								
Frequently	76,929	70.7	16,790	21.4	20.3–22.5			
Rarely/never	31,959	29.3	8237	25.9	24.3–27.6	1.20	1.15–1.25	<0.001
**Parents check the child’s homework**								
Frequently	33,500	31.0	6661	19.4	18.0–20.9			
Rarely/never	74,756	69.0	18,256	24.3	23.2–25.5	1.25	1.19–1.30	<0.001
**Parents are understanding of the child’s problems**								
Frequently	47,895	44.1	9495	19.4	18.0–20.9			
Rarely/never	60,674	55.9	15,492	25.4	24.4–26.5	1.31	1.26–1.36	<0.001
**Main carers rummaging without permission through their child’s personal belongings**								
Rarely/never	91,579	84.4	20,569	22.1	20.9–23.3			
Frequently	16,868	15.6	4393	25.9	24.3–27.6	1.18	1.13–1.22	<0.001
**Physical aggression committed by an adult in the family**								
No	97,010	89.4	20,740	21.0	19.8–22.3			
Yes	11,470	10.6	4233	36.8	34.9–38.7	1.73	1.67–1.80	<0.001
**PHYSICAL ENVIRONMENT**								
**Tobacco use in the family**								
None of the parents/guardians	76,809	72.5	16,989	21.8	20.4–23.3			
At least one	29,160	27.5	7317	24.6	23.7–25.4	1.12	1.07–1.18	<0.001
**PSYCHOSOCIAL INDICATORS**								
**Feeling of loneliness**								
Rarely/never	90,204	83.2	19,051	20.7	19.6–21.9			
Frequently	18,254	16.8	5972	32.4	30.6–34.3	1.53	1.48–1.59	<0.001
**Loss of sleep due to concerns**								
Rarely/never	97,873	90.4	21,303	21.4	20.2–22.6			
Frequently	10,447	9.6	3683	34.7	33.0–36.3	1.59	1.55–1.64	<0.001
**Existence of close friends**								
3 or more friends	87,401	80.6	19,304	21.7	20.4–23.0			
Less than 3 friends	20,983	19.4	5707	26.8	25.5–28.2	1.23	1.18–1.28	<0.001
**Child’s truancies**								
Rarely/never	105,722	97.2	23,961	22.3	21.1–23.6			
Frequently	3092	2.8	1070	34.4	32.6–36.2	1.56	1.45–1.68	<0.001
**LIFESTYLE**								
**Daily time of sedentary leisure**								
≤4 h a day	67,733	62.3	14,427	20.9	19.6–22.1			
>4 h a day	40,966	37.7	10,558	25.8	24.3–27.4	1.22	1.17–1.27	<0.001
**TV watching**								
≤2 h a day	41,120	37.8	8832	21.0	19.5–22.6			
>2 h a day	67,672	62.2	16,178	23.6	22.5–24.8	1.11	1.06–1.16	<0.001
**Tobacco use (ever in life, even one or two puffs)**								
No	86,113	79.1	17,979	20.6	19.3–21.9			
Yes	22,784	20.9	7056	31.3	30.6–32.0	1.56	1.47–1.64	<0.001

^1^ weighted proportion and 95% CI.s. ^2^ regressions adjusted by sex and age (STEP 1 of the analysis sequence).

**Table 2:** Asthma Symptoms in Brazilian Adolescents Characteristics Studied, Intra-Block and Final Analysis. PeNSE survey 2012.

*n*: Total 108,350, with Asthma Symptoms 25,038, 23.1% (Weighted % 22.7, 21.5–23.9)	PR ^1^	95% CI	*p*-Value	PR ^2^	95% CI	*p*-Value
**DEMOGRAPHIC CHARACTERISTICS**						
**Gender**						
Boys						
Girls	1.17	1.08–1.27	<0.001			
**Age**						
<14 years						
14 years	1.00	0.96–1.04	0.10			
15 years	0.97	0.93–1.01				
>15 years	0.92	0.85–1.00				
**SOCIOECONOMIC CONDITIONS ^3^**						
**SES indicator**						
1st tertile + 2nd tertile (D + C class)						
3rd tertile (B class)	1.15	1.09–1.22	<0.001	1.15	1.09–1.22	<0.001
**School’s administrative status**						
Public						
Private	1.07	1.00–1.13	0.037	1.07	1.00–1.13	0.037
**EXPOSURE TO VIOLENCE**						
**Feeling safe on the way from home to school**						
Yes						
No	1.19	1.14–1.25	<0.001	1.15	1.05–1.17	<0.001
**Feeling safe at school**						
Yes						
No	1.37	1.30–1.45	<0.001	1.29	1.18–1.30	<0.001
**Being victim of bullying**						
Rarely/never						
Frequently	1.36	1.29–1.44	<0.001	1.29	1.10–1.24	<0.001
**Exposure to fights with firearms**						
No						
Yes	1.63	1.55–1.70	<0.001	1.48	0.71–1.75	<0.001
**FAMILY CONTEXT AND DYNAMICS**						
**Parents are frequently present at home**						
Presence of mother and/or father						
Absence of mother and father	1.09	1.00–1.18	0.05			
**Parents are aware of the leisure activities of their child**						
Frequently						
Rarely/never	1.04	1.00–1.09	0.05			
**Parents are present during meals**						
Frequently						
Rarely/never	1.11	1.06–1.51	<0.001	1.09	1.05–1.14	<0.001
**Parents check the child’s homework**						
Frequently						
Rarely/never	1.13	1.08–1.19	<0.001	1.13	1.08–1.18	<0.001
**Parents are understanding of the child’s problems**						
Frequently						
Rarely/never	1.19	1.13–1.25	<0.001	1.18	1.13–1.23	<0.001
**Main carers rummaging without permission through their child’s personal belongings**						
Rarely/never						
Frequently	1.18	1.13–1.24	<0.001	1.14	1.09–1.19	<0.001
**Physical aggression committed by an adult in the family**						
No						
Yes	1.61	1.56–1.67	<0.001	1.38	1.33–1.43	<0.001
**PHYSICAL ENVIRONMENT**						
**Tobacco use in family**						
No						
Yes	1.12	1.07–1.18	<0.001	1.09	1.04–1.15	0.001
**PSYCHOLOGICAL ASPECTS**						
**Feeling of loneliness**						
Rarely/never						
Frequently	1.38	1.33–1.43	<0.001	1.23	1.18–1.27	<0.001
**Loss of sleep due to concerns**						
Rarely/never						
Frequently	1.37	1.33–1.41	<0.001	1.20	1.16–1.24	<0.001
**Existence of close friends**						
3 or more friends						
Less than 3 friends	1.15	1.10–1.19	<0.001	1.09	1.05–1.13	<0.001
**Child’s truancies**						
Rarely/never						
Frequently	1.45	1.34–1.57	<0.001	1.09	1.02–1.18	0.009
**LIFESTYLE**						
**Daily time of sedentary leisure**						
≤4 h sitting watching TV etc./day						
> 4 h sitting watching TV etc./day	1.17	1.13–1.21	<0.001	1.08	1.05–1.11	<0.001
**Daily time of TV watching**						
≤2 h/day						
> 2 h/day	1.04	1.00–1.08	0.048	1.04	1.01–1.08	0.015
**Tobacco personal use**						
No						
Yes	1.53	1.45–1.61	<0.001	1.27	1.22–1.33	<0.001

^1^ STEP 2: intra-block regressions on asthma symptoms of the variables whose *p*-values were ≤0.10 in tri-variate analysis (STEP 1); adjusted by sex, age and all the variables in the block. ^2^ STEP 3: regression on asthma symptoms adjusted by age, sex and the variables whose *p*-value was ≤0.05 in STEP 2, with further exclusion of variables whose *p*-value was >0.05 at intra-level analysis. ^3^ mothers’ and fathers’ education are not included in this stage of analysis, because they are component of the economic indicator.
